# How progress evaluations are used in postgraduate education with longitudinal supervisor-trainee relationships: a mixed method study

**DOI:** 10.1007/s10459-022-10153-3

**Published:** 2022-09-12

**Authors:** Marnix P. D. Westein, A. S. Koster, H. E. M. Daelmans, M. L. Bouvy, R. A. Kusurkar

**Affiliations:** 1 Department of Pharmaceutical Sciences, Utrecht University, Universiteitsweg 99, 3584 CG Utrecht, The Netherlands; 2 Research in Education, Faculty of Medicine Vrije Universiteit, Amsterdam, The Netherlands; 3 The Royal Dutch Pharmacists Association (KNMP), The Hague, The Netherlands; 4 Programme Director Master of Medicine, Faculty of Medicine Vrije Universiteit, Amsterdam, The Netherlands

**Keywords:** Progress evaluation, Performance assessment, Postgraduate education, Supervisor, Feedback, Longitudinal relationship

## Abstract

The combination of measuring performance and giving feedback creates tension between formative and summative purposes of progress evaluations and can be challenging for supervisors. There are conflicting perspectives and evidence on the effects supervisor-trainee relationships have on assessing performance. The aim of this study was to learn how progress evaluations are used in postgraduate education with longitudinal supervisor-trainee relationships. Progress evaluations in a two-year community-pharmacy specialization program were studied with a mixed-method approach. An adapted version of the Canadian Medical Education Directives for Specialists (CanMEDS) framework was used. Validity of the performance evaluation scores of 342 trainees was analyzed using repeated measures ANOVA. Semi-structured interviews were held with fifteen supervisors to investigate their response processes, the utility of the progress evaluations, and the influence of supervisor-trainee relationships. Time and CanMEDS roles affected the three-monthly progress evaluation scores. Interviews revealed that supervisors varied in their response processes. They were more committed to stimulating development than to scoring actual performance. Progress evaluations were utilized to discuss and give feedback on trainee development and to add structure to the learning process. A positive supervisor-trainee relationship was seen as the foundation for feedback and supervisors preferred the roles of educator, mentor, and coach over the role of assessor. We found that progress evaluations are a good method for directing feedback in longitudinal supervisor-trainee relationships. The reliability of scoring performance was low. We recommend progress evaluations to be independent of formal assessments in order to minimize roles-conflicts of supervisors.

## Introduction

In assessment programs in healthcare education, individual assessments are used for feedback, and intermediate evaluations of the trainees’ progress are used to compare the performance to the given standard, and to contribute to the trainees’ development as a professional (St-Onge et al., 2020; Van Der Vleuten et al., 2012, 2015). The combination of measuring performance and giving feedback during these progress evaluations results in tension between formative and summative purposes (Watling & Ginsburg, 2019). Longitudinal supervisor-trainee relationships are advocated for giving meaningful feedback and creating supportive environments (Bowen et al., 2015; Ramani et al., 2020; Voyer et al., 2016). However, there are conflicting perspectives and evidence on the effects of supervisor-trainee relationships on assessing performance. These relationships can make assessments either more accurate or increase rater bias (de Jonge et al., 2017; Lee & Ross, 2020; Schut et al., 2021). For supervisors, it can be challenging to combine the (potentially) conflicting roles of educator and assessor (Govaerts et al., 2019; Jackson et al., 2019; Schut et al., 2021). In this study, the validity of progress evaluations for measuring trainee performance and the utility for supporting trainee development was investigated using a multifaceted approach.

As competency-based education has become commonplace in postgraduate healthcare education, implementation of assessment programs has become a new frontier. Various instruments have been developed to measure performance of trainees on required competencies and roles (St-Onge et al., 2020; Yaqoob Mohammed Al Jabri et al., 2021). Programmatic assessment, the integration of assessment *for* learning and assessment *of* learning, has been developed to enable meaningful triangulation of datapoints for robust decision-making and has been found to catalyse learning (Schut et al., 2021). Progress evaluations are designed to report on progression towards competency of trainees, and to be informative in its value for learning (St-Onge et al., 2020; Van Der Vleuten et al., 2012, 2015). Trainees as well as their supervisors consider progress evaluations as a tool for providing feedback, in the sense that it provides explicit descriptions of observed performance (St-Onge et al., 2020). Performance measurement is also considered valuable for monitoring progress, but the tension between formative and summative assessment purposes, can hamper learning opportunities (Govaerts et al., 2019; Schut et al., 2021).

Supervision can be defined as the provision of monitoring, guidance, and feedback on matters of personal, professional, and educational development in the context of patient care (Kilminster & Jolly, 2000). In order to contribute to the learning, assessment, and patient safety goals of the educational program, supervisors take up multiple roles as educator, assessor, mentor, and coach (Lee & Ross, 2020; Mellon & Murdoch-Eaton, 2015; Sawatsky et al., 2020; Schut et al., 2021). These roles can strengthen each other, but can also be conflicting. Supervisors often have to combine formative and summative assessment purposes, be efficient and effective, and meet the needs of learners and education institutes (Govaerts et al., 2019). In their role as assessor, supervisors can for example feel disinclined to provide honest or critical feedback because they fear the impact of feedback on learners (Schut et al., 2021). Navigating the multiple roles is essential for a successful supervisor-trainee relationship (Jackson et al., 2019).

Investment in prolonged and trustworthy teacher-learner relationships is one of the proposed strategies to improve the value of assessment programs (Schut et al., 2021). In postgraduate healthcare education, supervisors and trainees often only work together for a brief period of time, which can make the exchange of assessment information less effective (Schut et al., 2021). In primary care settings, such as the general practice and the community pharmacy, longitudinal supervisory relationships are more common (Jackson et al., 2019; Lee & Ross, 2020; Westein et al., 2019). Continuity of supervision has potential benefits for assessment: earlier identification of learners in difficulty, creating opportunities for trust, and providing greater patient care responsibilities (Lee & Ross, 2020). However, there are also potential risks of introducing bias, based on past performance or forward feeding, and increasing rater bias from having too few raters (Lee & Ross, 2020).

The purpose of this study was to learn how progress evaluations are used by supervisors in a longitudinal supervisor-trainee relationship. It was performed in a 2-year postgraduate Community-Pharmacy specialization program for pharmacists in the Netherlands, in which supervisors combine the roles of educator, assessor, and employer (Westein et al., 2019). Three research questions directed the study: a) What is the validity of the progress evaluation scores? b) What is the utility of the progress evaluations? (c) How does the supervisor-trainee relationship influence the use of progress evaluations?

## Methods

This study had a sequential explanatory mixed-method design, with quantitative data gathering and analyses followed by qualitative data gathering and analyses (Kajamaa et al., 2020). The study was granted ethical approval by the NVMO Ethical Review Board (record number 2018.6.9).

### Setting

In the Dutch postgraduate Community-Pharmacy Specialization program trainees are employed at the training pharmacy and supervised by a supervisor during the 2-year program. The specialization program uses the CanMEDS 2005 framework, in which the roles have been adapted to the community pharmacy setting: Pharmaceutical Expert, Communicator, Collaborator, Scholar, Health advocate, Manager, and Professional (Frank, 2005; Westein et al., 2019).

The supervisor takes part in a mandatory two-day training, in which the specialization program is explained and feedback and assessment skills are trained (Westein et al., 2019). In practice, the supervisor combines direct clinical supervision of the trainee with indirect supervision (the supervisor is available in the pharmacy or via phone) and oversight (the supervisor reviews the care afterwards) (Farnan et al., 2012). During the 2-year program, the supervisor gives structured feedback on 36 predefined Entrustable Professional Activities (EPAs) using several tools of which the results are noted in a digital portfolio (Westein et al., 2019). Both in year 1 and year 2, the supervisor performs three progress evaluations (see the progress evaluation form in appendix 1). The supervisor is also responsible for a summative performance evaluation at the end of year 1 and 2. In addition to the workplace-based learning and assessment, trainees take part in centralized courses and assignments.

The above-described program was introduced in 2012. Unlike most healthcare specializations in the Netherlands, no external funding is supplied. The availability of training positions is partly dependent on the employment market, and the supervisor is (as the employer) responsible for the continuation and discontinuation of the trainees’ employment contract. The program director of the specialization program is responsible for the decision making within the specialization program.

### Data collection and analyses

The progress evaluation scores of trainees, who started the specialization between Jan 2012 and Sept 2015, were extracted from their digital portfolios in July 2018, and anonymized. The level of performance on each CanMEDS role, and a general competence score were scored by supervisors on a 4-point scale (1 = insufficient, 2 = moderate, 3 = adequate, 4 = good). Progress evaluation scores were rated at six timepoints (3-months, 6-months, 9-months, 15-months, 18-months, 21-months). The reference point was the standard expected by the supervisor upon final completion of the two-year program. A repeated measures ANOVA was performed in SPSS 28 to assess the effects of time, and roles on the progress evaluation scores. As each trainee was trained and evaluated by a single supervisor in a unique pharmacy setting, it was not possible to analyse the inter-rater variance as a source of variance.

Between December 2018 and April 2019, 15 semi-structured interviews were held with supervisors to examine the inferences they made while scoring, and to explore their perceptions of the utility of the progress evaluations, and their view on how the supervisor-trainee relationship might have influenced the progress evaluations. The first researcher developed a set of questions for the interviews and corresponding codes for the data analysis using Kane’s validity framework, and the concept of programmatic assessment (see appendix 2) (Kane et al., 1999; Schuwirth & van der Vleuten, 2012; Van Der Vleuten et al., 2012). Two research team members gave feedback on the questions and codes. The set of questions and codes were finalized in the full research team. Supervisors whose trainees had successfully completed their training within six months prior to the supervisors’ interview were selected for this study. They were approached by email and telephone to join the study. In order to prepare for the interview, the participants received the questions in advance through email. The first researcher interviewed the participants by telephone at a predetermined date and time. Interviews were transcribed verbatim and anonymized. Directed content analysis was used to assign the data to the predetermined codes (Hsieh & Shannon, 2005). Two researchers independently coded the first two interviews and discussed the results afterwards. The first researcher then conducted and coded the remaining interviews (based on the agreed strategy) by using Atlas.ti 8. This first researcher identified themes and discussed them within the team to identify subthemes.

## Results

In this section, we first report on the characteristics of trainees whose assessment data was included in the study, characteristics of the supervisors who were interviewed, and descriptive statistics. Next, we report on the validity evidence for the measured progress evaluation scores, the utility of the progress evaluations, and the influence of supervisor-trainee relationships on progress evaluations.

### General results

#### Characteristics of the trainees

At the moment of data extraction, from the 342 trainees who had started their training in the given period, 304 (89%) had completed it, 23 (7%) trainees had officially discontinued their training, and 15 (4%) trainees were still in training. Most trainees were female (*n* = 237, 69%). On average, the trainees were 27 years old at the beginning of their training, ranging from 24 to 42 years old.

#### Characteristics of the interviewed supervisors

Fifteen supervisors were interviewed to capture their experiences with progress evaluations. The telephone interviews took on average 50 min (range 35–72 min). Thirty-two supervisors had been approached, 9 supervisors were not willing to participate in the interviews (‘lack of time’ was given most frequently as a reason), and 8 supervisors could not be reached. There were six interviewees who had supervised a trainee for their first time, and nine interviewees who had supervised one or more trainees prior to their last trainee in the current or previous curriculum. The gender and experience of the interviewed supervisors were representative for the supervisor population.

#### Descriptive statistics

Appendix 3 shows the descriptive statistics of the progress evaluation scores (1 to 4) at six timepoints (T = 3, 6, 9, 15, 18, 21) for the seven CanMEDS roles and the general competence.

When we examined the scores on the CanMEDS roles, the score of ‘1’ was never given for the roles of collaborator and scholar, and rarely for the other roles (≤ 1%). At 3-months of training, the median score for each role was ‘3’. The lowest mean score at that moment was for the manager role (mean (SD) = 2.78 (0.65)) and highest mean score was for the collaborator role (mean (SD) = 3.22 (0.62)). At 21-months of training, the median score for each role was ‘4’. The lowest mean score was still for the manager role (mean (SD) = 3.58 (0.55)) and highest mean score was for the pharmaceutical expert role (mean (SD) = 3.89 (0.31)). The mean score on the 7 CanMEDS roles at each timepoint did not significantly differ from the mean general competence score.

### Validity evidence

Quantitative and qualitative data were analysed to investigate the degree to which validity and reliability evidence supported the use of progress evaluation scores.

#### Reliability / precision

**Fig. 1:**
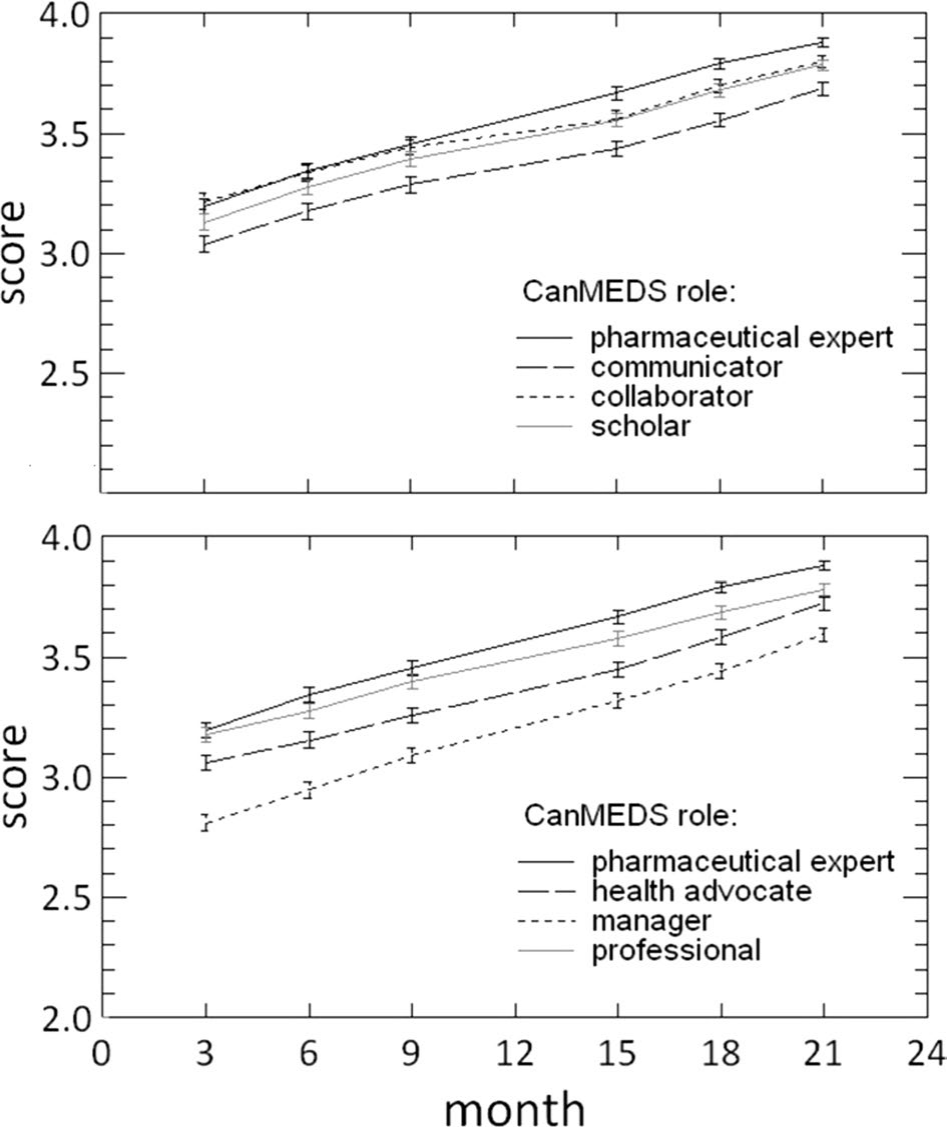
Mean progress evaluations scores of trainees in time for each role (± standard error of the mean). Data were collected at 3, 6, 9, 15, 18 and 21 months. Scores are given on a 4-point scale (1 = insufficient, 2 = moderate, 3 = adequate, 4 = good)

The repeated measures ANOVA of the progress evaluation scores showed that the scores were significantly affected by time (F_3,36_ = 253.73, *P* < 0.001), the role (F_4,48_ = 63.2, *P* < 0.001), and the interaction of time and role (F_23,31_ = 2.231, *P* < 0.001). Mauchly's Test of Sphericity indicated that the assumption of sphericity had been violated, and therefore, a Greenhouse–Geisser correction was used. As shown in Fig. 1, the mean scores for each role increased in time. Compared to the other roles, the Manager role scored relatively low.

#### Response processes

The interviews with supervisors showed that the response processes of supervisors varied in relation to the intended interpretation of scores. First of all, supervisors struggled with the subjective aspect of evaluating performance. They had difficulty objectifying competence and found it difficult to estimate their own strictness or lenience when inferring scores.

“I do not know if I am competent to assess a trainees’ performance, other than following my gut feelings. I cannot objectify. That is my weakness.. ..so I would like to learn how to objectify, so that it is not all guesswork.” (supervisor 07)

Secondly, most supervisors interpreted the 4-point scale as if it was a 3-point scale. They considered a score of ‘2’ as performance below average, but with improvement in range, a score of ‘3’ as performance above the minimum requirement, but without excelling, and a score of ‘4’ as performance at a good or very good level, with no improvement needed. These scores were in the common range for a trainee. Supervisors associated a score of ‘1’ with serious performance or attitudinal issues, and considered it to be a disqualification of the trainees’ competence. In turn, this reflected negatively on their own supervising skills. Therefore, some supervisors acknowledged that they refrained from assigning a score of ‘1’.

“I would actually never say performance is insufficient. Moderate means that performance could be a lot better. Adequate means, well, we are on the right track, and good means we have arrived where we want to be.” (supervisor 08)

Thirdly, supervisors thought it was more important that the progress evaluation scores reflected (and stimulated) performance growth, rather than actual performance. Many supervisors said they consciously lowered scores from a ‘4’ to a ‘3’ in year 1 as to keep room for improvement during the latter part of the program. It was also argued that scoring all ‘4’s’ early in training would be demotivating for trainees.

“In the beginning you try to score a bit lower, because otherwise the training would be finished in a month, while you are not ready yet. ..over the course of the second year we scored the roles as adequate (= ‘3’) as much as possible..” (supervisor 03)

Scoring a ‘2’ was considered acceptable in year 1, while in year 2 scores of ‘3’ and ‘4’ were expected of the trainee. When development was absent or the trainee displayed no intentions to grow, supervisors considered the performance to be problematic.

### Utility of the progress evaluations

#### Structuring the education program

Supervisors appreciated the 3-monthly progress evaluations for the structure it brought in the 2-year program: (1) to evaluate and score trainee performance, (2) to look back at the progress made in the previous period, and (3) to discuss the planning and activities for the upcoming period. In addition, supervisors valued it as a practical reminder for trainees to update their digital portfolios. All supervisor agreed on a frequency of once every three months for the progress evaluations. Evaluating more often would give too much repetition, and less often would result in losing touch with trainees’ progress. Notes were collected by supervisors and trainees in the progress evaluation reports, and often the personal development plan would be adjusted afterwards.

#### Opportunity for feedback and coaching

Some supervisors used the progress evaluations for coaching conversations. Trainees were given the opportunity to actively seek feedback. Supervisors and trainees together reached agreement on the goals and tasks for the upcoming period. Some supervisors also considered aspects other than the progress made at the training location, such as trainees’ performance in centralized courses and trainees’ personal situation.

“..those conversations during progress evaluations go into depth. And yes, they sometimes took really long.. ..maybe it is my style of supervision, but you are more a psychologist, then a supervising pharmacist. So then you discuss [with the trainee]..’ how do you approach things, where do you run into, how are things at home, because you have a fulltime position, but you also have other things to do, how do you do these things..’ Well, those sort of discussions.” (supervisor 13)

Some supervisors stressed that motivation and case discussion were more important than scores, as in their view feedback was the primary goal of progress evaluations.

“I think those scores are like a ritual dance.. ..I just give [the trainee] my feedback.” (supervisor 04)

#### Predictive properties

Supervisors considered trainee performance in the first year to be a good indicator for the performance in the second year. Some supervisors expected trainees’ strengths to keep developing and trainees’ weaknesses to stay behind in development. However, other supervisors had experienced an (unexpected) positive change in trainees’ development in the second year, either due to changes in the pharmacy context or due to trainees’ personal changes.

“I had a trainee of whom I had doubts after the first year, but she made up for it in the second year. ..I think that she was very modest in the beginning, and stayed in the background. Eventually, an experienced colleague left, and she got the opportunity to fill that position, and that is what she did. ..it turned out very well.” (supervisor 06)

### Supervisor-trainee relationship

Supervisors were asked how their relationship with the trainee affected the progress evaluations. We found that supervisors considered a positive bond, to be the foundation for feedback, scaffolding, and support. The bond was described as the ability to trust each other, and to be open to feedback, and a mutual willingness to learn. A positive bond enhanced the value of progress evaluations as it led to a more reliable impression of trainee performance, and more honest feedback. A positive bond was also reflected in the scores for the role of collaborator.

“You really need to build a good bond, in order to sit down together and tell each other the blunt truth. That is something even some couples do not do, so you really need to connect.” (supervisor 13)

Other important aspects of a good relationship where having shared goals, and clear agreements. Several supervisors emphasized the importance of selecting a trainee to train in their pharmacy with personal attributes that agreed with their own, and who fitted in the community pharmacies’ local culture. A poor relationship, was considered to give a less reliable picture of the trainees’ performance, and made giving (negative) feedback more difficult.

Within the longitudinal relationship supervisors found it difficult to help trainees whose development stagnated. They were uncertain how to put the trainee on the right track, how to stimulate trainees in developing their qualities and attributes that kept behind, and how to make them learn from mistakes.

“Sometimes in the second year.. someone develops a certain competency even further, while the competencies which the trainee needs to develop, stay behind. And that is a bit.. I find that a bit difficult, I need more experience in that.” (supervisor 01)

Some supervisors mentioned that power imbalance between supervisor and trainee affected the relationship negatively. The imbalance was further accentuated when the supervisor was also the trainee’s employer.

### The educational environment

Some supervisors described the supervisor-trainee relationship as part of a larger context of creating a safe educational environment at the workplace. Supervisors described three factors for creating such an environment: (1) the attitude and behaviour of the supervisor (to be fallible and open to critique, meanwhile displaying self-confidence and keeping a positive mindset, to establish a clear hierarchy in the organization, to support the trainee in difficult situations, and have frequent direct contact with the trainee with opportunities for discussion), (2) the attitude and behaviour of the team (for them to be self-confident and willing to give feedback, to be helpful towards each other and towards trainees / students in the pharmacy, and to not feel threatened by them, and to be enthusiastic about teaching and learning in general), and (3) the physical and mental space for the trainee (having an own desk, having autonomy to undertake new tasks, and to organize their tasks as they see fit, to have a clear position within the pharmacy as licensed pharmacist, and to feel safe to express weaknesses and make mistakes).

“..to feel safe towards me, but also.. ..that I told him ‘you are not infallible, you are allowed to say it when you do not know something or are unable to perform a task.’ And if that was the case, that we would look at it again, place him in a situation in which he could learn that competency.. ..Further, I made it very clear to the pharmacy team, ‘the trainee is a licensed pharmacist. I am not very hierarchical, but he is higher than you in the hierarchy. So when I am not here and he makes a decision that you are not happy with, his decision still stands.” (supervisor 04)

## Discussion

Progress evaluations have a developmental function and an informative function in relation to the performance standard (St-Onge et al., 2020; Tromp et al., 2012; Van Der Vleuten et al., 2012). In our study, we found evidence for a statistically significant increase of progress evaluation scores over time. In addition, statistically significant differences between CanMEDS role scores were observed. The interviews with supervisors showed that supervisors varied widely in their response processes for scoring performance, thereby introducing an additional source of variance, and reducing the reliability of the progress evaluation scores for comparing trainees. From a supervisor perspective, the developmental function of the progress evaluations clearly preceded the informative function to the performance standard. Supervisors were willing to adjust the progress evaluation scores to motivate their trainees. In addition, we found that a positive supervisor-trainee relationship strengthened the feedback function of progress evaluations. In their longitudinal relationship, supervisors preferred the roles of educator, mentor, and coach over the role of assessor.

### Validity of the progress evaluation scores

The progress evaluation scores increased in time and were also dependent on the CanMEDS role to which they were assigned. Dory et al. have shown that perceptions of assessors influence the reliability of scoring instruments (Dory et al., 2018). In our study, supervisors struggled with the subjectivity of the assessments they performed, and some supervisors deliberately lowered progress evaluation scores in the first year as a prompt for growth in the second year, thereby introducing a strong rater bias. As every trainee within the specialization program was trained, and evaluated by a single supervisor in a single pharmacy, the reliability for comparing progress evaluation scores was unknown.

Training of supervisors is valuable and necessary and it takes time to implement an assessment program (Kilminster & Jolly, 2000; Schut et al., 2021). The supervisors in our study did receive training and instructions on scoring trainees. However, given the responses of supervisors this was not sufficient for reaching a uniform approach in measuring performance during progress evaluations.

Trainee performance on the CanMEDS roles was graded using a 4-point Likert scale and a fixed-reference point. As has been reported in previous studies, we found a reluctance amongst supervisors to classify trainee performance as inadequate (Barrett et al., 2016; Gingerich et al., 2020). Supervisors rarely gave the lowest score of ‘1’, and they expressed that this score was only to be used for trainees with serious performance or attitudinal issues. Some supervisors even refrained from assigning the score of ‘1’. Recently, the term underperformance has been introduced to normalize struggling behaviour of trainees (Gingerich et al., 2020). Based on our findings, we argue that underperformance should not be rated on a regular performance scale. Instead a ‘blame-free’ handling of underperformance should be enabled, which balances the expectancy of reporting poor performance, its’ value and costs for both supervisor and trainee, as suggested by Mak-van der Vossen (Mak-van der Vossen, 2019).

### Utility of progress evaluations

Progress evaluations are valuable for supervisors as well as trainees for making their expectations explicit and through facilitating feedback on areas of strength and improvement (St-Onge et al., 2020; Tromp et al., 2012; Van Der Vleuten et al., 2012). As described in the model of programmatic assessment, progress evaluations can also be valuable for the program as a whole for monitoring and predicting progress and identifying trainees who need remediation (St-Onge et al., 2020; Tromp et al., 2012; Van Der Vleuten et al., 2012). In our study, supervisors generally found the performance in the first year to be a good indicator for the second year performance. For supervisors, the main goals of progress evaluations was to discuss and give feedback on trainee development and to add structure to the learning process. Weallans, et al. have developed a composite model for providing effective feedback in clinical supervision (Weallans et al. 2022). In correspondence with this model, supervisors in our study reported that during progress evaluations feedback could be given based on past performance, and trainees could be challenged to seek self-assessment. Additionally, it was found that there was room for coaching conversations in which trainees’ views were explored, and goals and actions for the upcoming period were shared and noted. Monitoring the level of performance was used as a tool for trainee guidance rather than for accountability on a program level.

The evidence of effectiveness for offering grades as part of giving feedback has been disputed (Lefroy et al., 2015a, 2015b). Grades are a powerful tool, and need to be explained to help trainees create meaning from them (Lefroy et al., 2015a, 2015b). It has been argued that grades might even be best avoided as some trainees will stop trying to learn when their grades are sufficient and some trainees will give up learning when their grades are poor (Lefroy et al., 2015a, 2015b; Lefroy et al., 2015a, 2015b; Schut et al., 2021). Current formal assessment systems may also disadvantage learners who have the courage to embrace their weaknesses as well as those learners who choose to hide their weaknesses (Sawatsky et al., 2020). Indeed some supervisors in our study said they would rather refrain from scoring during progress evaluations and focus completely on giving feedback.

### Supervisor-trainee relationship

The supervisor-trainee relationship has been described as the most important factor for the effectiveness of supervision, more important than the supervisory methods used (Kilminster & Jolly, 2000). Based on Bordin’s ‘working alliance-based model of supervision’, Jackson et al. identified factors influencing this relationship: (1) the quality of the bond, (2) agreement on goals, tasks of supervision, and roles in supervision, (3) clarity and openness, (4) personal attributes of supervisor and trainer, and (5) quality of the local (and wider) educational environment (Jackson et al., 2019). In the interviews with supervisors, we found each of these factors. Telio et al. found a strong relation between trainees’ credibility judgements on the supervisor-trainee relationship and their interpretations of supervisory feedback (Telio et al., 2016). In our study, supervisors considered having a good bond as a foundation for feedback, scaffolding, and support, and they preferred to employ trainees in their pharmacy with personal attributes that fitted their own. The progress evaluations facilitated making clear agreements on goals and tasks of supervision for the upcoming period. During progress evaluations a good bond led to more open and honest feedback. A safe educational environment was described as a prerequisite. Our study also confirmed the importance of longitudinal supervisor-trainee relationships for giving meaningful, developmental feedback (Bowen et al., 2015; Lee & Ross, 2020; Lefroy et al., 2015a, 2015b; Ramani et al., 2020; Schut et al., 2021; Watling & Ginsburg, 2019).

### Conflicting roles of the supervisor

In our study, supervisors were responsible for the daily clinical supervision of trainees, for 3-monthly progress evaluations, and for summative performance evaluations at the end of year 1 and 2. During progress evaluations they had to combine the roles of assessor (scoring performance), educational supervisor (supporting agenda setting and planning (Mellon & Murdoch-Eaton, 2015)), mentor (guiding individual development and re-examination of their development (Mellon & Murdoch-Eaton, 2015)), and coach (orientating towards growth, nurturing reflection, and using failure as a learning opportunity (Sawatsky et al., 2020)). Combining these roles leads to tension, as formal assessment can set up the trainee as a performer and the supervisor as the audience for the trainees’ performance (Daelmans et al., 2016; Govaerts et al., 2019; Lee et al., 2019; Mellon & Murdoch-Eaton, 2015; Sawatsky et al., 2020). Trainees may stage a performance to portray confidence. Moreover, supervisors may feel disinclined to provide honest or critical feedback, fearing the impact of the feedback on trainees (Schut et al., 2021). In our study, supervisors had difficulty integrating assessment *for* learning with assessment *of* learning. Most supervisors saw their primary role as mentor and coach rather than as assessor.

Power imbalance is considered a potential threat to the ability of the supervisors and trainees for reaching agreement. The combination of the supervisor’s assessor and monitor roles and hierarchical relationships increase this power imbalance (Jackson et al., 2019). In the community pharmacy program in the Netherlands, supervisors are also the trainees’ employers. This power imbalance was described by supervisors as affecting the supervisor-trainee relationship negatively. Clarity of the power differential, transparency in the hierarchy, and trust between supervisor and trainee are key (Castanelli et al., 2022; Falender & Shafranske, 2017; Mohtady et al., 2019). The power imbalance requires further attention in the community pharmacy specialization program and also in other programs with a similar structure.

## Limitations

A major limitation of this study was the inability to quantify the inter-rater variance between supervisors. As the specialization program is arranged with every trainee having a single supervisor, an experimental study design in which trainees and supervisors are not nested, is needed (Moonen-van Loon et al., 2013). To ensure a level of supervisory experience amongst the interviewees, we selected supervisors for the interviews, who had successfully trained at least one trainee, and were willing to join the study. This could have resulted in a selection bias, in which the perceptions of supervisors with less or with negative supervisory experiences were excluded. Another limitation is the absence of the trainee perspective in the study. Since a shared understanding between supervisors and trainees of the nature and purpose of assessments is needed for assessment to be effective (Schut et al., 2021), we advise researching trainee perspective on the value of progress evaluations.

## Conclusions

We found 3-monthly progress evaluations to be a good method for directing feedback in postgraduate healthcare education. In contrast to programmatic assessment models, we found limited value of progress evaluations for making summative judgements. Positive supervisor-trainee relationships are a foundation for feedback, scaffolding, and support, and longitudinal relationships aide developmental feedback. To facilitate supervisors in their roles as mentor and coach, we recommend progress evaluations to be independent of formal decisions. The removal of grading from the progress evaluations can contribute to the authenticity of direct observations and the resulting feedback, and foster a culture of improvement.

## Progress evaluation form

- Date of activity
- CanMEDS roles (score 1 = insufficient, 2 = moderate, 3 = adequate, 4 = good)
  o Pharmaceutical expert
  p Communicator
  q Collaborator
  r Scholar
  s Health Advocate
  t Manager
  u Professional
  v General competence
- Trainee development
  o Specific strong learning experience (free text)
  p Specific learning experience to improve (free text)
  q Agreed approach for improvement (free text)
- Points of discussion
  o Is the trainee able to work in accordance with the personal development plan? (Yes/no)
  p Is the portfolio kept up to date by the trainee? (Yes/no)
  q How satisfied is the trainee with the educational environment? (free text)
- Advices and agreements
  o Advices and agreements about adjusting the personal development plan (free text)

## Interview guide—Questions related to progress evaluations

1. Describe your experience as a supervisor in the guidance and assessment of trainees
  - How long have you been guiding trainees?
  - When did you finish your supervisor training?
2. How do you feel about scoring trainees on the CanMEDS roles?
  - Do you feel that you are sufficiently capable to judge a trainee?
  - Can you give a concrete example of what you find difficult or easy?
  - How did you learn to assess a trainee?
  - Which competencies or roles are easy and which are hard to assess and what are the reasons?
3. Progress evaluations
  - Can you explain how you determine the scores when assessing a trainee?
  - Which rules do you apply when scoring the CanMEDS roles?
  - On which sources of information do you base these scores?
  - What role do Entrustable Professional Activities play in reaching these scores?
  - What are the arguments that you use to underpin these scores? Where do you record the arguments?
  - Do you have a norm or reference score in year 1 and year 2 of the program? If so, what is this norm / reference score?
  - In what way do the trainee and other people involved influence the given scores?
  - Do you feel that your relation with the trainee influences the scores? If so, how?
  - Do you feel comfortable and in the position to give the lowest score (= 1) and/or highest score (= 4) to the roles / trainee?
  - When a trainee performance below average on a certain aspect, what score would you give?
  - What is your opinion on the frequency of the 3-monthly progress evaluations? Would you like to do these more or less often? If so, why?
  - Besides scoring the CANMEDS roles, you are also asked to give an general competence score? How do you infer this score? In what way is it different than the average score on the seven CanMEDS roles?
  - Do the scores on the seven CanMEDS roles and the general competence score give a complete picture of the trainees’ performance as a community pharmacist at the given moment? Can you elaborate on this?
  - In what way do the given scores influence the training of the trainee?
  - During progress evaluations you are also asked to note positive aspects and aspects for improvement. How would you place the scores on the CanMEDS roles within the overall feedback to the trainee during an progress evaluations?
  - What do the scores given in year 1 tell you about the further performance of the trainee within the program?

## Appendix 3: Descriptive statistics of the progress evaluation scores

**Table:**

Role	N	Minimum	Maximum	Mean (SD)	Median
*Measurements at 3-months*					
Pharmaceutical expert	320	1	4	3.19 (0.55)	3
Communicator	322	1	4	3.02 (0.65)	3
Collaborator	321	2	4	3.22 (0.62)	3
Scholar	319	2	4	3.12 (0.59)	3
Health advocate	316	2	4	3.06 (0.56)	3
Manager	316	1	4	2.78 (0.65)	3
Professional	321	1	4	3.17 (0.58)	3
General competence	317	2	4	3.10 (0.50)	3
*Measurements at 6-months*					
Pharmaceutical expert	311	2	4	3.34 (0.54)	3
Communicator	312	2	4	3.18 (0.60)	3
Collaborator	312	2	4	3.35 (0.57)	3
Scholar	311	2	4	3.28 (0.55)	3
Health advocate	310	1	4	3.16 (0.57)	3
Manager	308	1	4	2.93 (0.63)	3
Professional	311	1	4	3.28 (0.55)	3
General competence	305	2	4	3.19 (0.49)	3
*Measurements at 9-months*					
Pharmaceutical expert	306	2	4	3.45 (0.52)	3
Communicator	307	2	4	3.29 (0.60)	3
Collaborator	308	2	4	3.45 (0.55)	3
Scholar	306	2	4	3.40 (0.57)	3
Health advocate	306	2	4	3.26 (0.55)	3
Manager	307	1	4	3.09 (0.59)	3
Professional	306	2	4	3.41 (0.54)	3
General competence	299	2	4	3.30 (0.49)	3
*Measurements at 15-months*					
Pharmaceutical expert	303	2	4	3.66 (0.48)	4
Communicator	303	2	4	3.45 (0.54)	3
Collaborator	304	2	4	3.58 (0.55)	4
Scholar	304	2	4	3.55 (0.52)	4
Health advocate	303	2	4	3.45 (0.53)	3
Manager	303	2	4	3.32 (0.58)	3
Professional	304	2	4	3.59 (0.52)	4
General competence	296	2	4	3.54 (0.51)	4
*Measurements at 18-months*					
Pharmaceutical expert	297	3	4	3.79 (0.41)	4
Communicator	296	2	4	3.56 (0.54)	4
Collaborator	296	2	4	3.70 (0.48)	4
Scholar	296	2	4	3.68 (0.47)	4
Health advocate	296	2	4	3.59 (0.50)	4
Manager	296	2	4	3.44 (0.55)	3
Professional	296	2	4	3.69 (0.48)	4
General competence	293	3	4	3.67 (0.47)	4
*Measurements at 21-months*					
Pharmaceutical expert	288	3	4	3.89 (0.31)	4
Communicator	291	2	4	3.70 (0.47)	4
Collaborator	290	2	4	3.80 (0.42)	4
Scholar	291	2	4	3.79 (0.43)	4
Health advocate	290	3	4	3.73 (0.44)	4
Manager	291	1	4	3.58 (0.55)	4
Professional	290	2	4	3.80 (0.43)	4
General competence	285	2	4	3.82 (0.40)	4

Progress evaluation scores of trainees collected at 3, 6, 9, 15, 18, and 21 months. Scores are given for each role on a 4-point scale (1 = insufficient. 2 = moderate. 3 = adequate. 4 = good). N = number of scores, SD = Standard deviation.
